# Effects of Obesity and Diabesity on Ventricular Muscle Structure and Function in the Zucker Rat

**DOI:** 10.3390/life12081221

**Published:** 2022-08-11

**Authors:** Ahmed Sultan, Ernest Adeghate, Bright Starling Emerald, Muhammad A. Qureshi, Saeed Tariq Minhas, Frank Christopher Howarth

**Affiliations:** 1 Department of Physiology, College of Medicine & Health Sciences, UAE University, Al Ain P.O. Box 17666, United Arab Emirates; 2 Department of Anatomy, College of Medicine & Health Sciences, UAE University, Al Ain P.O. Box 17666, United Arab Emirates

**Keywords:** heart, ventricular myocytes, shortening, intracellular Ca^2+^, ventricular muscle structure, mitochondria, sarcomere, Zucker fatty rat, Zucker diabetic fatty rat, Zucker lean rat

## Abstract

(1) Background: Cardiovascular complications are a leading cause of morbidity and mortality in diabetic patients. The effects of obesity and diabesity on the function and structure of ventricular myocytes in the Zucker fatty (ZF) rat and the Zucker diabetic fatty (ZDF) rat compared to Zucker lean (ZL) control rats have been investigated. (2) Methods: Shortening and intracellular Ca^2+^ were simultaneously measured with cell imaging and fluorescence photometry, respectively. Ventricular muscle protein expression and structure were investigated with Western blot and electron microscopy, respectively. (3) Results: The amplitude of shortening was increased in ZF compared to ZL but not compared to ZDF myocytes. Resting Ca^2+^ was increased in ZDF compared to ZL myocytes. Time to half decay of the Ca^2+^ transient was prolonged in ZDF compared to ZL and was reduced in ZF compared to ZL myocytes. Changes in expression of proteins associated with cardiac muscle contraction are presented. Structurally, there were reductions in sarcomere length in ZDF and ZF compared to ZL and reductions in mitochondria count in ZF compared to ZDF and ZL myocytes. (4) Conclusions: Alterations in ventricular muscle proteins and structure may partly underlie the defects observed in Ca^2+^ signaling in ZDF and ZF compared to ZL rat hearts.

## 1. Introduction

As of 2021, there were around 573 million adults between the ages of 20–79 who were suffering from diabetes mellitus (DM), an illness characterized by high blood glucose levels, and this number is expected to increase to 643 million by 2030 and around 783 million by the year 2045 [1]. Since 1975, obesity, defined as “abnormal or excessive fat accumulation that may impair health”, has almost tripled. Globally, more than 1.9 billion adults aged 18 and older were overweight, and 650 million were obese in 2016 [2]. Diabetes and obesity are risk factors for cardiovascular disease, which is the main cause of morbidity and mortality in these patients [3]. Those who are obese are at greater risk of developing type 2 diabetes (T2DM). In this condition, the body may produce and release sufficient insulin into the bloodstream, but the cells become resistant to the effects of insulin. The term “diabesity” was created due to the strong link between obesity and diabetes [4,5].

Recently, scientists developed a novel model of T2DM and obesity called the Zucker diabetic fatty (ZDF) rat [6]. ZDF rats were derived from the Zucker fatty (ZF) (fa/+) male rats, which genetically inherit obesity as an autosomal Mendelian recessive trait. The ZF rat has a missense mutation (fatty, fa) in the leptin receptor gene (Lepr) that leads to hyperphagia and the development of obesity without DM. The third group, the ZDF homozygous (fa/fa) male rats, developed DM as early as 10 weeks of age, reaching 100% incidence by 21 weeks of age [7]. As such, it is an ideal model to understand how obesity-induced T2DM can lead to diabetic cardiomyopathy (DCM), which is a major adverse complication of T2DM characterized by defects in both systolic and diastolic function. 

Contractile and Ca^2+^ signaling defects in the heart vary according to the selected experimental model of obesity and diabetes [8]. The aim of this study was to investigate the effects of obesity and diabesity on the function and structure of ventricular myocytes and muscle tissue in the Zucker fatty (ZF) rat and the Zucker diabetic fatty (ZDF) rat compared to Zucker lean (ZL) control rats.

## 2. Materials and Methods

### 2.1. Animals

**Figure 1:**
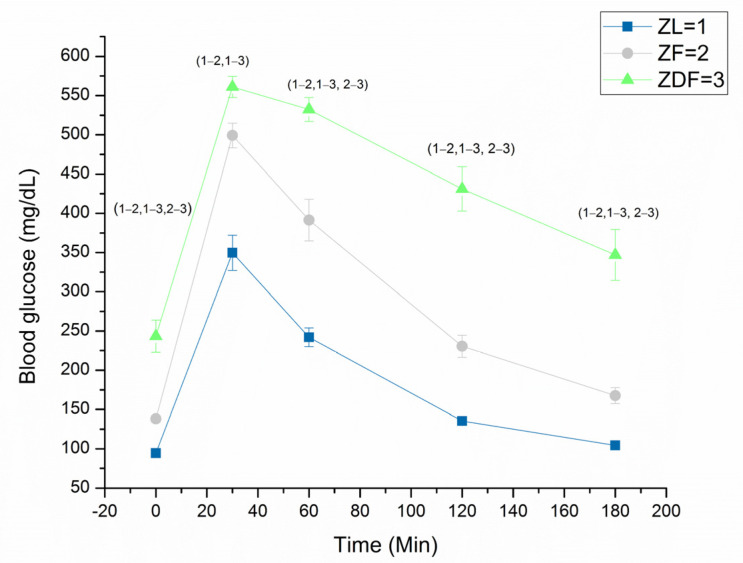
Glucose tolerance test. Data are mean ± SEM, *n* = 20 ZL, 18 ZF and 19 ZDF rats. Mean rank at 0 min is [10.65] ZL, [31.44] ZF and [46.0] ZDF, at 30 min [12.33] ZL, [31.94] ZF and [43.76] ZDF, at 60 min [12.5] ZL, [30.06] ZF and [45.37] ZDF, at 120 min [11.2] ZL, [30.83] ZF and [46.0] ZDF, and at 180 min [10.7] ZL, [31.5] ZF and [44.65] ZDF. Numbers in parenthesis represent significant differences.

Experiments were performed in 20 ZDF, 21 ZF and 21 ZL male rats aged 195 days (6.5 months) (Charles River Laboratories, Margate, Kent, UK). Rats were maintained under a 12-h light/12-h dark cycle with free access to water and standard rat diet. Heart weight and body weight as well as non-fasting blood glucose (OneTouch Ultra 2, LifeScan, Milpitas, CA, USA) measurements were made immediately before experiments. Glucose tolerance tests (GTTs) were performed just before the start of the experiments. Briefly, after an overnight fast, the fasting blood glucose was collected and measured from the tail vein of non-anaesthetized rats. Animals were then injected according to their body weights with glucose (2 g kg^−1^ body weight, intraperitoneal), and blood glucose was measured from tail venous blood at 30, 60, 120 and 180 min after glucose injection (Figure 1). Following sacrifice, blood was collected in EDTA filled glass tubes and centrifuged at 400 RPM (C2 series, Centurion Scientific, Stoughton, UK) for 5 min, and the supernatant blood plasma was removed and stored at −80 °C for Insulin ELISA measurements. Ethical consent for this research was obtained from the Animal Ethics Committee, College of Medicine & Health Sciences, United Arab Emirates University, Al Ain, UAE.

### 2.2. Isolation of Ventricular Myocytes

Left ventricular myocytes were isolated from the rats according to previously described techniques [9]. In brief, the animals were euthanized using a guillotine and hearts removed rapidly and mounted for retrograde perfusion according to the Langendorff method. Hearts were constantly perfused at a flow rate of 8 mL g heart^−1^ min^−1^ and at 36–37 °C with cell dissociation solution containing in mmol/L: 5.4 KCl, 0.75 CaCl_2_, 1.4 MgCl_2_, 130 NaCl, 20 taurine, 0.4 NaH_2_PO_4_, 10 creatine, 5.0 HEPES and 10 glucose (pH 7.3). Perfusion flow rate was adjusted to allow for differences in heart weight between animals. When the heart had stabilized, perfusion continued for 4 min with Ca^2+^-free cell dissociation solution comprising 0.1 mmol/L EGTA and then for 6 min with cell dissociation solution comprising 0.075 mg/mL type XIV protease (Sigma, Taufkirchen, Germany), 0.75 mg/mL type 1 collagenase (Worthington Biochemical Corp, Lakewood, NJ, USA) and 0.05 mmol/L Ca^2+^. The left ventricle tissue was removed from the heart, chopped and gently shaken in a dissociation solution containing collagenase supplemented with 1% BSA. The cells were filtered from this solution at intervals of 4 min and reused in a cell dissociation solution with 0.75 mmol/L Ca^2+^. 

### 2.3. Simultaneous Measurement of Ventricular Myocyte Shortening and Intracellular Ca^2+^

Ventricular myocyte shortening and intracellular Ca^2+^ were simultaneously measured in Fura-2 AM loaded myocytes according to previously described techniques [9]. Myocytes were loaded with the fluorescent indicator Fura-2 AM (F-1221, Molecular Probes, Eugene, OR, USA). In brief, 6.25 µL of a 1.0 mmol/L stock solution of Fura-2 AM (dissolved in dimethylsulphoxide DMSO) was added to 2.5 mL of cells to give a final Fura-2 concentration of 2.5 µmol/L. Myocytes were shaken gently for 10 min at room temperature (24 °C). After loading, myocytes were centrifuged, washed with normal Tyrode solution to remove extracellular Fura-2 and then left for 30 min to ensure complete hydrolysis of the intracellular Fura-2 ester. Ventricular myocytes were allowed to settle on the glass bottom of a Perspex chamber mounted on the stage of an inverted Axiovert 35 microscope (Zeiss, Göttingen, Germany). The cells were perfused with normal Tyrodes (3–5 mL/min) containing the following in mmol/L: 1.8 CaCl_2,_ 1.0 MgCl_2_, 5 KCl, 5 HEPES, 140 NaCl and 10 glucose (pH 7.4) at 35–36 °C. Shortening was measured in electrically stimulated (1 Hz) ventricular myocytes with an IonOptix MyoCam imaging system (IonOptix Corporation, Milton, MA, USA). Time to peak (TPK) shortening, resting cell length (RCL), amplitude (AMP) of shortening and time to half (THALF) relaxation were measured. Data were acquired and analyzed with IonWizard 6.6 version 10 software (IonOptix LLC, 396 University Ave. Westwood, MA, USA).

To assess intracellular Ca^2+^ concentration, the Fura-2 loaded myocytes were interchangeably lit by 340 and 380 nm light utilizing a monochromator (Cairn Research, Faversham, UK) that changed the excitation light every 2 ms. The subsequent fluorescence emitted at 510 nm was collected by a photomultiplier tube, and the fraction of the emitted fluorescence at the two (340/380 ratio) excitation wavelengths was estimated to provide an index of intracellular Ca^2+^ concentration. TPK Ca^2+^ transient, resting Fura-2 ratio, AMP of Ca^2+^ transients and the THALF decay of the Ca^2+^ transients were measured in electrical field (1 Hz) stimulated myocytes maintained at 35–36 °C. Data were acquired and analyzed with IonWizard 6.6 version 10 software (IonOptix LLC, 396 University Ave. Westwood, MA, USA).

### 2.4. Myofilament Sensitivity to Ca^2+^

**Figure 2:**
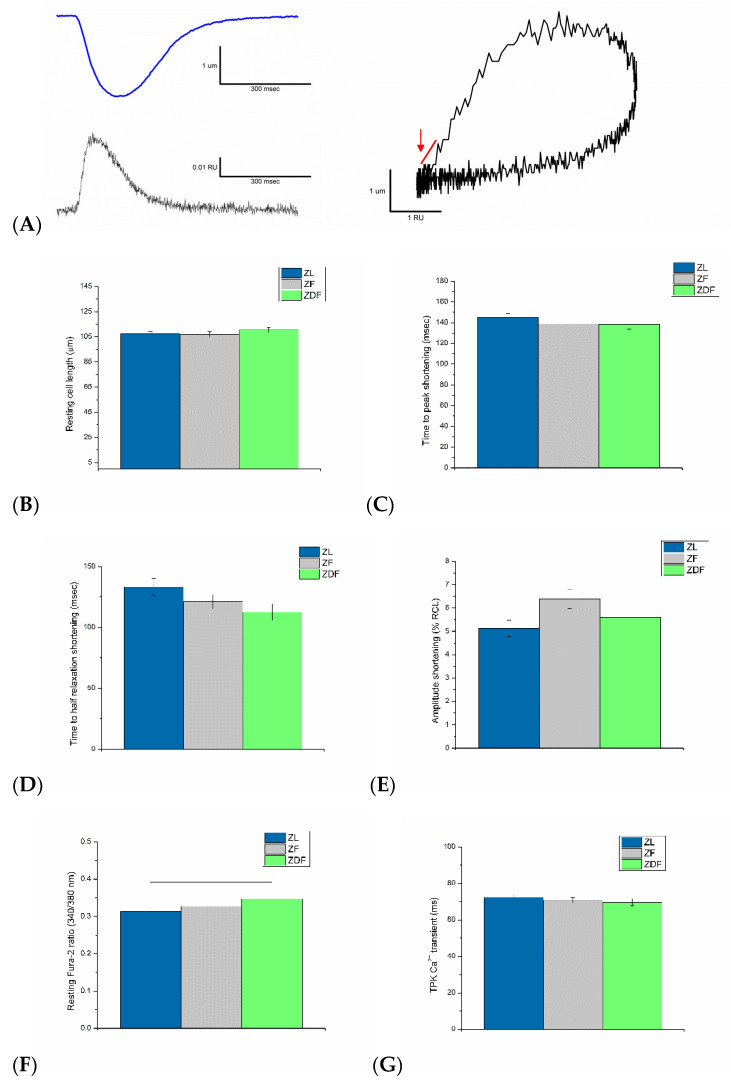

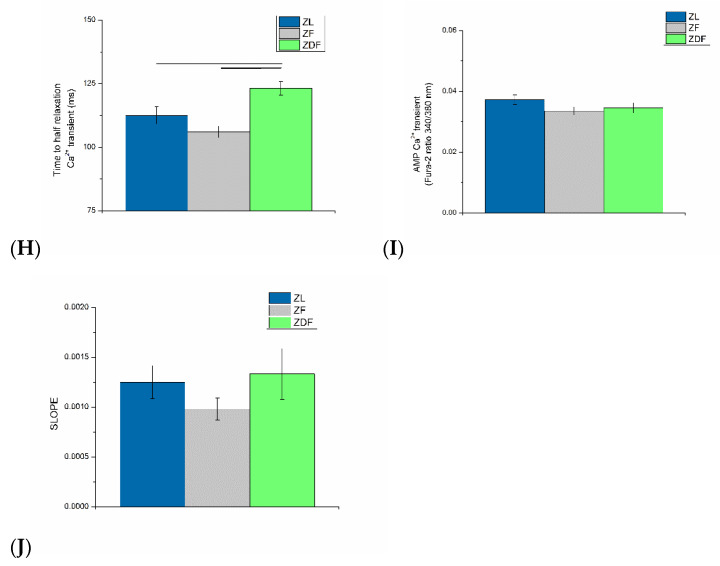
Simultaneous measurement of shortening and intracellular Ca^2+^ in electrically stimulated (1 Hz) ventricular myocytes. Typical simultaneous recording of shortening and Fura-2 ratio in a ventricular myocyte from a ZL rat ((**A**), left panel), typical phase–plane diagram of Fura-2 ratio versus cell length ((**A**), right panel). Resting cell length (**B**) time to peak (TPK) shortening, (**C**) time to half (THALF) relaxation of shortening (**D**), amplitude shortening (**E**), resting Fura-2 ratio (**F**), time to peak of the Ca^2+^ transients (**G**), time to half relaxation of the Ca^2+^ transients (**H**), amplitude of the Ca^2+^ transients (**I**). Mean gradient of the Fura-2 trajectory through late relaxation of the twitch contraction during the period (500–800 ms). The arrow shows the area that the gradient was measured (**J**). Data are mean ± SEM. *n* = 57 ZL, 65 ZF and 52 ZDF cells from 5 ZL, 5 ZF and 5 ZDF rats.

Myofilament sensitivity was measured according to previously described techniques [10]. In order to assess myofilament sensitivity, the Fura-2 ratio was plotted against shortening (Figure 2A, left panel). Myofilament sensitivity to Ca^2+^ was evaluated from the subsequent phase–plane diagram (as shown in Figure 2A, right panel) by calculating the gradient of the Fura-2 cell length trajectory in the course of late relaxation (500–800 ms) of the twitch contraction, as formerly described [11,12]. The position of the trajectory signifies the comparative myofilament response to Ca^2+^ and hence, can be used as a measure of myofilament sensitivity to Ca^2+^ [13].

### 2.5. Protein Assessment

Heart left ventricles were obtained from the rats after sacrifice. Tissue samples were flash-frozen in liquid nitrogen and stored at −80 °C for later use. After thawing, tissue extracts were prepared by homogenization on ice with RIPA lysis buffer (1× PBS, 50 mM NaF, 0.5% Na deoxycholate (*w*/*v*), 0.1% SDS, 1% IGEPAL, 1.5 mM Na3VO4, 1mM PMSF and 1× Halt) protease/phosphatase inhibitor cocktail, followed by centrifuging for 10 min at 3000–4000 r.p.m. at 4 °C. The supernatant was collected and quantitated with Barford’s assay using BSA (10 mg/mL stock) as standard. Western blot analysis was performed with 20–50 µg of nuclear, cytoplasmic, or total cellular protein extracts that was loaded in 4–12% Tris-HCl SDS-PAGE gels (Bio-Rad, Hercules, CA, USA) using MOPS running buffer, which contains Tris, Mops Sah, SDS and EDTA, and was transferred using a transfer buffer containing Trizm Base and glycine (Bienne, Switzerland) onto a PVDF membrane. Blocking was performed for 2 h at room temperature with 5% nonfat skimmed milk powder dissolved in TBST containing Tween 20, NaCl and Trizm Base for 1–3 h. Extracts were then incubated with primary and secondary antibodies. The following primary antibodies were used: glyceraldehyde-3-phosphate dehydrogenase (GAPDH) (ab8245, Abcam, Cambridge, UK), Myosin (ab50967, Abcam), Ryanodine (ab2868, Abcam), Sarcoplasmic reticulum calcium ATPase-2 (SERCA2) (MA3-919, ThermoFisher Scientific, Waltham, MA, USA), Tropomyosin (ab7785, Abcam), Troponin C (ab137130, Abcam), Troponin I (ab47003, Abcam), Troponin T (ab8295, Abcam) and Connexin (Cx45) (ab135474, Abcam). The following secondary antibodies were used: Anti-rabbit IgG HRP-linked antibody (7074S, Cell Signaling Technology, Danvers, MA, USA) and Anti-mouse IgG HRP-linked antibody (7076S, Cell Signaling Technology). The loading marker used was Kaleidoscope (#1610375, Bio-Rad). Stripping of the membrane was performed with a stripping buffer that contains Tris and SDS, followed by β-Mercaptoethanol incubation in a water bath at 60 °C for 30 min, followed by washing with TBST, then blocking and re-probing again. Gel images were scanned, and the signal intensity was quantified using ImageJ software (NIH) [14]. 

### 2.6. Ultrastructural Assessment

Transmission electron microscopy (TEM) was performed according to previously described techniques [15]. After sacrifice hearts were removed and left ventricles tissue samples were dissected from the same morphological area, free from other accessory tissues, they were cut into small pieces and washed with 0.1 M phosphate buffer and then immersed in a modified McDowell and Trump (1976) fixative for 3 h at room temperature. After rinsing with phosphate buffer, the tissues were postfixed with 1% osmium tetroxide for 1 h. The tissue samples were later washed with distilled water, dehydrated in a series of graded ethanol and propylene oxide and then infiltrated and embedded in Agar100 epoxy resin and polymerized at 65 °C for 24 h. Blocks were trimmed and semithin and ultrathin sections were cut with Leica EM UC7 Ultracuts ultramicrotome (Vienna, Austria). Semithin sections (1.5 µm thickness) were stained with 1% aqueous toluidine blue on glass slides. Ultrathin sections (95 nm) with gilded color were collected on 200 mesh copper grids and then were contrasted with uranyl acetate, followed by lead citrate. The grids were examined and photographed with a Tecnai Biotwin Spirit G2, Transmission Electron Microscope from the FEI company (Eindhoven, The Netherlands). Typical EM images in ventricular muscle from 5 ZL, 3 ZF and 5 ZDF groups of Zucker rats were analyzed per field at 6000× and 11,500× magnification. Images were also quantified using ImageJ software v.1.53n, Wayne Rasband and contributors, NIH, Bethesda, MD, USA. http://imagej.nih.gov/ij accessed on 13 June 2022 [14]. 

### 2.7. Statistical Evaluation

Results were expressed as the mean ± SEM of *n* observations. Statistical comparisons were performed by Origin 9.0 (OriginLab, Northampton, MA, USA) and IBM SPSS statistics software using either the Kruskal–Wallis H one-way ANOVA test followed by Bonferroni corrected *t* tests for multiple comparisons or independent samples *t* test, or paired samples *t* test, as appropriate. *p* values of less than 0.05 were considered to indicate a significant difference. 

## 3. Results 

### 3.1. General Characteristics

The general characteristics of the ZDF, ZF and ZL rats are shown in Table 1. The ZF and ZDF groups exhibited significantly increased body weight (H(2) = 34.93, *p* < 0.001) and heart weight (H(2) = 17.8, *p* < 0.001) compared to ZL rats. Non-fasting blood glucose (H(2) = 42.15, *p* < 0.001) was significantly elevated in ZDF (345.10 ± 25.16 mg/dL) rats compared to ZF (136.67 ± 4.38 mg/dL) and ZL (118.47 ± 4.18 mg/dL) rats, which were similar to previously reported results [6]. Non-fasting blood glucose was not significantly (*p* > 0.05) different between ZF and ZL rats. The ZF (3.26 ± 0.11 mg/g) rats showed a significant increase in heart weight/body weight ratio (H(2) = 22.12, *p* < 0.001) compared to ZDF and ZL rats. Heart weight/body weight ratio was also modestly higher in ZDF compared to ZL rats, which is consistent with the results of a previous study [16]. Insulin ELISA was measured using a commercially available rat insulin ELISA kit (10-1250-01, Mercodia rat Insulin ELISA, Uppsala, Sweden). ZF rats (12.70 ± 1.15 µg/L) had significantly (H(2) = 17.92, *p* < 0.001) higher insulin levels compared to ZL (2.09 ± 0.57 µg/L) and ZDF (2.14 ± 0.29 µg/L), and these results are similar to a previously published study [17]. 

### 3.2. Glucose Tolerance Test

The GTT was performed just before the experiments and the results are shown in Figure 1. The fasting blood glucose was highest in ZDF (243.47 ± 20.52 mg/dL, *n* = 19), intermediate in ZF (138.17 ± 4.43 mg/dL, *n* = 18) and lowest in ZL (94.40 ± 2.29 mg/dL, *n* = 20) rats, and these differences were significant at 0 min (H(2) = 44.79, *p* < 0.001), at 30 min (H(2) = 36.04, *p* < 0.001), at 60 min (H(2) = 38.36, *p* < 0.001) and at 120 min (H(2) = 43.16, *p* < 0.001). At 180 min following the glucose challenge, blood glucose remained significantly (H(2) = 42.55, *p* < 0.001) elevated in ZDF (346.94 ± 32.33 mg/dL, *n* = 17) and ZF (167.61 ± 10.15 mg/dL, *n* = 18) compared to ZL (104.4 ± 3.75 mg/dL, *n* = 20) rats. In a previous study [18], the GTT was performed only up to 60 min, whereas in this study GTT was performed up to 180 min, and that provides glucose recovery data over a longer period following glucose challenge. 

### 3.3. Ventricular Myocyte Shortening and Intracellular Ca^2+^

A typical record of electrically evoked shortening-2 ratio in a ZL myocyte is shown in Figure 2A (left upper panel). The mean resting cell length was not significantly altered in ZDF (110.50 ± 1.75 µm) or in ZF (107.04 ± 1.96 µm) compared to ZL (107.33 ± 1.66 µm) (Figure 2B). Neither the TPK shortening (Figure 2C) nor the THALF relaxation of shortening (Figure 2D) was significantly (*p* > 0.05) altered in ZDF or ZF compared to ZL controls. The AMP shortening (Figure 2E) was modestly higher (*p* > 0.05) in ZF (6.38 ± 0.41%RCL) compared to ZL (5.13 ± 0.35% RCL) but not compared to ZDF (5.60 ± 3.33% RCL) myocytes. 

A typical record of electrically evoked Ca^2+^ transient in a ZL myocyte is shown in Figure 2A (left lower panel). The mean resting Fura-2 ratio (340/380 nm) was significantly (H(2) = 9.56, *p* = 0.008) increased in ZDF (0.35 ± 0.008 RU) mean rank [104.35] compared to ZL (0.31 ± 0.007 RU) mean rank [74.83] in myocytes (Figure 2F). The mean TPK Ca^2+^ transient was not significantly (*p* > 0.05) different between ZF (70.86 ± 1.42 ms), ZDF (69.69 ± 1.92 ms) and ZL (72.42 ± 1.84 ms) myocytes (Figure 2G). THALF decay of the Ca^2+^ transient was significantly (H(2) = 26.26, *p* < 0.001) prolonged in ZDF (123.23 ± 2.68 ms) mean rank [115.66] compared to ZL (112.54 ± 3.46 ms) mean rank [84.03] and shortened in ZF (106.08 ± 2.30 ms) mean rank [68.02] compared to ZDF myocytes (Figure 2H). AMP of the Ca^2+^ transient was not significantly (*p* > 0.05) different between ZF (0.034 ± 0.0013 RU), ZDF (0.035 ± 0.0017 RU) and ZL (0.037 ± 0.0016 RU) myocytes (Figure 2I). 

### 3.4. Myofilament Sensitivity to Ca^2+^

Records of shortening and Ca^2+^ transient were used to create a phase–plane diagram. A typical phase–plane diagram of Fura-2 ratio versus cell length in a ZL myocyte is shown in Figure 2A (right panel). The mean gradient of the Fura-2 cell-length trajectory calculated throughout the late relaxation periods of the twitch contraction (500–800 ms) is shown in Figure 2J. The gradient was not significantly (*p* > 0.05) different in ZDF and ZF compared to ZL myocytes. 

### 3.5. Protein Assessment

**Figure 3:**
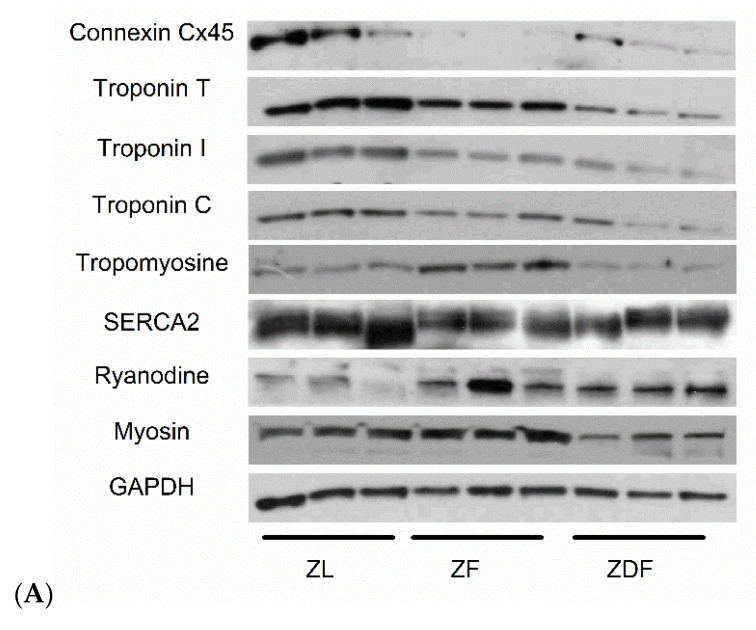

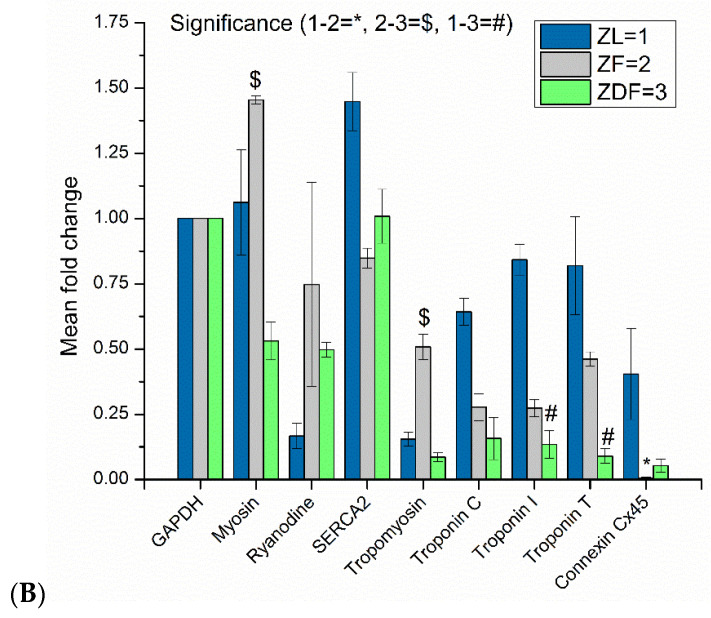
Assessment of proteins in ventricular muscle. Original Western blot gels (**A**) and mean fold changes (**B**). Error bars represent mean ± SEM. GAPDH was used as the loading control (*n* = 3–6 hearts).

Western blot analysis revealed significant differences in the expression of proteins that are involved in the process of cardiac muscle contraction. Typical blots are shown in Figure 3A. The mean fold changes in protein expression are shown in Figure 3B. GAPDH was used as loading control. Myosin expression was significantly (H(2) = 7.2, *p* = 0.027) lower in ZDF (0.53 ± 0.07) mean rank [2.0] compared to ZF (1.45 ± 0.02) mean rank [8.0] but not compared to ZL (1.06 ± 0.2) mean rank [5.0] ventricle tissue. Interestingly, Ryanodine expression was only modestly elevated (*p* > 0.05) in ZDF and ZF compared to ZL ventricle tissue and SERCA2 expression was modestly (*p* > 0.05) lower in ZDF (1.01 ± 0.1) and ZF (0.85 ± 0.04) compared to ZL (1.45 ± 0.11) ventricle tissue. Tropomyosin expression was significantly (H(2) = 6.49, *p* = 0.039) higher in the ZF (0.51 ± 0.05) mean rank [8.0] group compared to ZDF (0.09 ± 0.02) mean rank [2.33] and ZL (0.16 ± 0.03) mean rank [4.67] controls. Troponin C expression was modestly lower (*p* > 0.05) in ZDF (0.16 ± 0.08) and in ZF (0.28 ± 0.05) compared to ZL (0.64 ± 0.05) ventricle tissue. Expression of Troponin I was significantly (H(2) = 6.49, *p* = 0.039) reduced in ZDF (0.13 ± 0.05) mean rank [2.33] compared to ZL (0.84 ± 0.06) mean rank [8.0] and Troponin T was significantly (H(2) = 6.49, *p* = 0.039) reduced in ZDF (0.09 ± 0.03) mean rank [2.0] compared to ZL (0.82 ± 0.19) mean rank [7.67] ventricle tissue. Connexin Cx45 was significantly (H(2) = 7.2, *p* = 0.027) reduced in ZF (0.007 ± 0.004) mean rank [2.0] compared to ZL (0.4 ± 0.17) mean rank [8.0] ventricle tissue.

### 3.6. Ultrastructural Assessment

**Figure 4:**
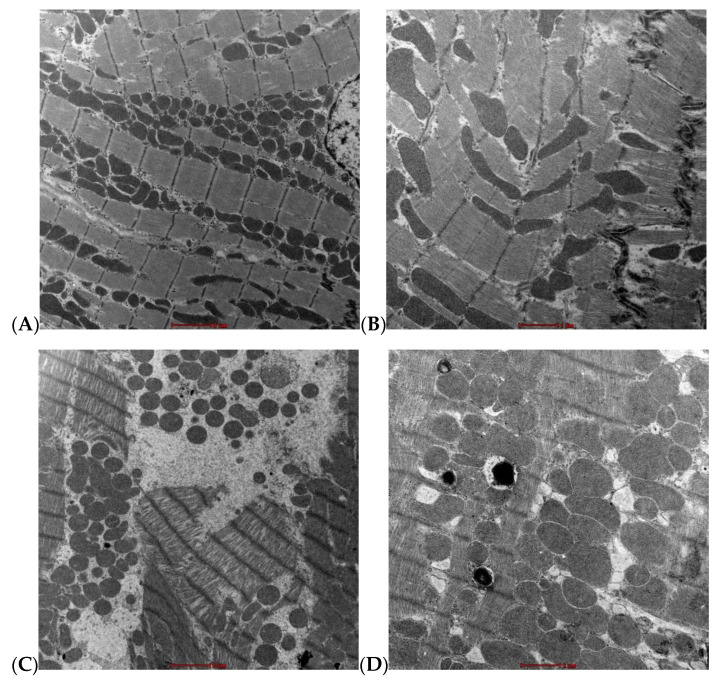

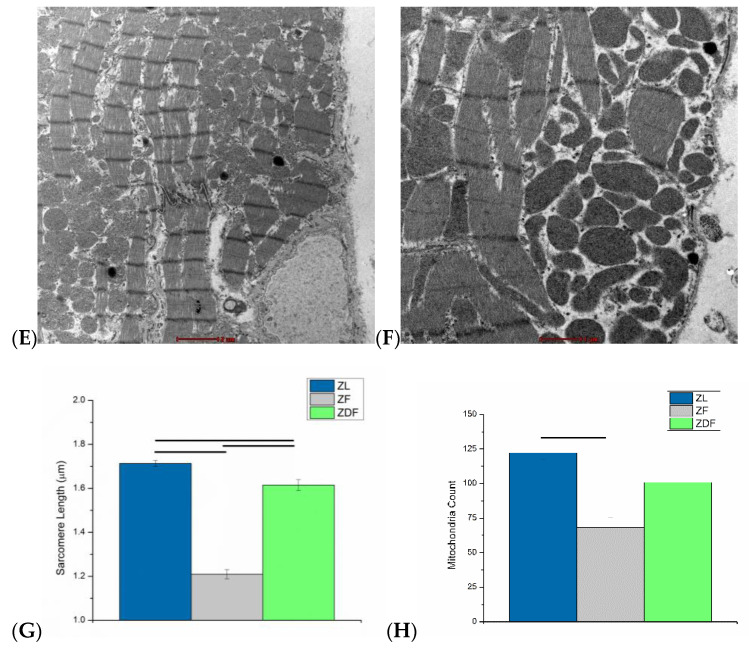
Assessment of structure in ventricular muscle. Typical transmission electron microscope (TEM) images from a ZL rat per field at 6000× magnification (**A**), and 11,500× magnification (**B**). Typical TEM images from a ZF rat per field at 6000× magnification (**C**), and 11,500× magnification (**D**). Typical TEM images from a ZDF rat at 6000× magnification (**E**), and 11,500× magnification (**F**). Mean Sarcomere length (**G**) and Mean mitochondria count (**H**). Data are mean ± SEM. *n* = 78 ZL, 33 ZF and 39 ZDF sections for Sarcomere length and *n* = 26 ZL, 13 ZF and 16 ZDF for Mitochondrial count all from 5 ZL, 3 ZF and 5 ZDF rats.

Morphometric analysis is presented in Figure 4. Typical TEM images in ventricular muscle from a ZL rat at 6000× magnification are shown in Figure 4A, and 11,500× magnification in Figure 4B. Typical TEM images in ventricular muscle from a ZF rat at 6000× magnification are shown in Figure 4C and 11,500× magnification in Figure 4D. Typical TEM images in ventricular muscle from a ZDF rat at 6000× magnification are shown in Figure 4E and 11,500× magnification in Figure 4F. Mean Sarcomere length is shown in Figure 4G and mean Mitochondria count in Figure 4H. Sarcomere length (H(2) = 82.93, *p* < 0.001) was shortest in ZF (1.21 ± 0.02 µm) mean rank [17.45], intermediate in ZDF (1.61 ± 0.03 µm) mean rank [76.41] and longest in ZL (1.71 ± 0.01 µm) mean rank [99.6] myocytes (Figure 4G). Mitochondria count (H(2) = 18.40, *p* < 0.001) was lowest in ZF (86.23 ± 7.26) mean rank [12.85], intermediate in ZDF (100.81 ± 10.50) mean rank [27.09] and highest in ZL (122.15 ± 4.45) mean rank [36.13] myocytes with significant difference (*p* < 0.001) between ZL and ZF (Figure 4H).

Qualitative image analysis showed that ZL sarcomeres are well oriented with structured dark bands and intercalated disks that are clearly visible, and the mitochondria have normal shape.

In ZF heart the sarcomeres were disorganized, dislocated, widened with spaces in between; mitochondria are clogged, deformed and small, lipid droplets are present.

Furthermore, ZDF heart sarcomeres are not as well organized as in ZL, but better than in ZF heart. Spaces were present in ZDF but not as severe as in ZF heart, lipid droplets are present and mitochondria are damaged as well as deformed. 

## 4. Discussion

Interestingly, blood insulin levels were significantly higher in ZF rats than they were in ZL and ZDF rats. ZDF rats were previously found to have elevated fasting serum glucose, insulin, cholesterol and triglyceride levels as well as impaired glucose tolerance [19]. Analyzing the signaling pathway of insulin might explain this. ZF rats could have a compromised ratio between bound and unbound insulin. In the first step of insulin signaling, insulin binds to its tyrosine kinase receptor the insulin receptor kinase (IRK); following autophosphorylation, the insulin–receptor complex is then internalized into the endocytic system (ENS), where glucose transporters are recruited from intracellular stores in skeletal and cardiac muscle and adipose tissue, which activates the metabolic process. After that, degradation of insulin is controlled by intra-endosomal acidification, in which “insulinase” stimulates the separation of insulin from the IRK. This degradation may also be compromised in the ZF rat [20,21]. The body weights of the ZDF rats are modestly lower than ZF rats. Insulin resistance in diabesity reduces the ability to transport glucose from the blood into the body’s cells to use as energy, and this results in a shift from carbohydrate to fat/protein metabolism, which in turn leads to a reduction in overall body weight. Electrically evoked myocyte shortening and intracellular Ca^2+^ were recorded simultaneously. The mean resting cell length of shortening was not significantly altered in ZDF compared to ZL and ZF myocytes. The mean TPK shortening as well as THALF relaxation of shortening were also not altered in ZDF compared to ZF and ZL myocytes. However, the AMP shortening was modestly increased in ZF compared to ZL but not compared to ZDF myocytes, which could be a sign of hypertrophy. Ca^2+^/calmodulin-dependent protein kinase II-delta (CaMKIIδ), a mediator of cardiac contractility, plays an important role in cardiac muscle contraction and relaxation as well as in the performance of the diabetic heart and is linked to cardiac hypertrophy [22,23,24,25,26]. CaMKIIδ may play a role in ZF myocyte Ca^2+^ signaling and Ryanodine receptor (RyR) SR Ca^2+^ release [26]. In a previous study, in rats aged 30–34 weeks of age, ZDF myocytes showed preserved THALF relaxation and AMP of shortening compared to ZL myocytes [12], whereas according to another study, the AMP of shortening at 6 and 22 weeks remained unchanged and the time course at 6 weeks did not differ. Nevertheless, the TPK shortening as well as the THALF relaxation of shortening were more pronounced in ZDF rats at 22 weeks [27]. 

Fura-2 ratio (340/380 nm) was significantly increased in ZDF compared to ZL myocytes. The mean TPK Ca^2+^ transient was not significantly different between groups. THALF decay of the Ca^2+^ transient was significantly prolonged in ZDF compared to ZL and was shortened in ZF compared to ZDF myocytes. AMP of the Ca^2+^ transient was not significantly different between groups. Contraction and relaxation are mainly regulated by intracellular Ca^2+^. During the process of Ca^2+^-induced Ca^2+^ release there is a small entry of Ca^2+^ through L-type Ca^2+^ channels and this small entry of Ca^2+^ triggers a large release of Ca^2+^ from the SR [28]. Research in different experimental models of diabetes has reported enhanced diastolic SR Ca^2+^ leakage, lower caffeine-evoked Ca^2+^ release and decreased rate of SR Ca^2+^-ATPase (SERCA)-mediated Ca^2+^ uptake in myocytes from db/db diabetic mice [29,30] as well as decreased AMP of caffeine-releasable Ca^2+^, SR store and release rates of Ca^2+^ and depressed resequestration into SR in myocytes from streptozotocin (STZ)-induced diabetic rats [31]. In type 2 diabetic patients, db/db mice, STZ and alloxan-induced diabetic rats, previous studies have shown that reduced Ca^2+^ release may result from structural defects in the SR Ca^2+^ release channel as well as decreased expression of Ca^2+^ release channel mRNA [32,33,34,35]. The SR also consists of longitudinal tubules, which release Ca^2+^ ions, and the terminal cisternae, which are large regions in close proximity to the ends of the transverse tubules known as “T-tubules” that sequester and concentrate Ca^2+^. Evidence suggests that loss of T-tubule integrity can profoundly affect excitation–contraction coupling (ECC) in myocytes, which is evident during action potential propagation [36,37,38]. It is important to note that T-tubule density can be measured using whole-cell capacitance in voltage-clamped myocytes relative to cell area; previous results indicate that rat myocytes can be kept in quiescent culture for 24 h with no detectable de-tubulation or loss of T-tubules, which is why we use freshly isolated myocytes [36]. Our results show that there may be a defect in the SR Ca^2+^ signaling in the ZDF group.

The Fura-2 cell length trajectory during the late stages of relaxation of the twitch contraction was not significantly different in myocytes between groups, which indicates that myofilament sensitivity to Ca^2+^ is not altered. Myofilament activation is modulated by protein phosphorylation, and myofilament proteins are substrates for PKC, PKA and CAMK, which in turn can induce changes in myofilament sensitivity to Ca^2+^ [39]. We have investigated certain proteins that might affect myofilament sensitivity to Ca^2+^, and although no changes have been found in Fura-2 length trajectory, we have found changes in expression of proteins of Troponin I and T proteins between groups, which may have a bearing on myofilament sensitivity to Ca^2+^. Our previously published paper reported that diabetic Goto-Kakizaki (GK) rat Fura-2 cell length trajectory was steeper in GK compared to control myocytes, which suggested an increase in myofilament sensitivity to Ca^2+^ [40]. 

Ventricular muscle protein was assessed with Western blot. Myosin expression was modestly lower in ZDF compared to ZF ventricle tissue, which is consistent with a previous study [41]. Myosin was significantly higher in ZF and lower in ZDF compared to GAPDH expression, and this was evident in TEM sections that show ZF alterations. Ryanodine expression was modestly increased in ZDF and ZF compared to ZL ventricle tissue; this might be a compensatory mechanism to preserve SR Ca^2+^ release [42,43]. Interestingly, SERCA2 expression was modestly lower in ZDF and ZF compared to ZL ventricle tissue. Expression of SERCA protein has been variously reported in different studies [44,45,46]. As previously mentioned, SERCA2 is important for SR Ca^2+^ handling. Tropomyosin expression was higher in ZF compared to ZDF and ZL ventricle tissue. Tropomyosin has been previously linked to hypertrophic cardiomyopathy [47,48]. In this experiment, we found evidence of structural defects in ZF rat that can be interpreted as signs of hypertrophy. Troponin C, Troponin I and Troponin T are very important in Ca^2+^ signaling and ECC coupling [39], and they also show the same trend with minor differences. Troponin C expression was modestly lower in ZDF and in ZF compared to ZL ventricle tissue. Expression of Troponin I and Troponin T were reduced in ZDF compared to ZL ventricle tissue. These myofilament proteins have been reported to be linked to dilated cardiomyopathy [49], and diabetes is also associated with dilated cardiomyopathy [50]. In this study, however, low expressions indicate that contraction and relaxation may be affected, possibly due to lack of force or dilatation in the muscle which can be observed in the TEM sections in the ZF and ZDF groups. Finally, gap junction protein Connexin Cx45 is important for electrical signal conduction between cardiac myocytes and heart rhythm [51]. In this experiment, the expression of Cx45 was reduced in ZF and modestly reduced in ZDF compared to ZL ventricle tissue, which may have implications for disturbances in heart rhythm, which have been reported in a previous study [52]. Interestingly, it has been reported that mRNA expression levels for Gja7 (Cx45) was significantly increased in SAN from diabetic heart compared to controls [53]. 

Morphometric analysis of the sections indicated that sarcomeres are longest in ZL, intermediate in ZDF and shortest in ZF myocytes. Similarly, the mitochondrial count is highest in ZL, intermediate in ZDF and lowest in ZF myocytes, with significant differences between ZL and ZF. The differences in mitochondrial count may reflect differences in size or metabolic activity between the groups. These morphological changes in mitochondria and signs of injury in the ZDF have also been similarly described in previous diabetic studies [50,54], which indicates that cardiomyopathy is a common feature of obesity and diabetes. Additionally, energy utilization has been associated with diabetes [55], and reductions in mitochondria could be signs of altered energy utilization and attributed to a more sedentary lifestyle in ZF as well as ZDF rats and their increased body weights. Furthermore, diabetes has been associated with heart failure (HF), and metabolic abnormalities have been reported extensively [56,57]. Also, mitochondrial reactive oxygen species are a major source of chronic oxidative stress in HF. They are also responsible for causing the electrical instability that leads to Sudden Cardiac Death [58]. 

## 5. Conclusions 

To our knowledge, this is the first study to investigate the effects of obesity and diabesity in the ZDF and ZF compared to ZL controls with simultaneous measurement of myocyte shortening and intracellular Ca^2+^ while identifying protein and structural defects. We have found evidence of molecular and structural defects in the ZF and ZDF rat heart. Our results suggest that SR Ca^2+^ handling, as well as energy utilization, is compromised in ZDF as well as in ZF. Myocyte contraction and relaxation may also be affected in the ZDF and ZF rat due to muscle protein structural defects, which lead to an affected heart rhythm and poor cardiac function. 

### 5.1. Study Limitations and Future Direction

The results of this study provide a strong foundation on which to develop functional and structural studies to further understand the effects of obesity and diabesity on electro-mechanical dysfunction in the Zucker rat heart. The number of rats was limited by the availability of grant funding, and experiments were conducted at a single time point. It would have been interesting to carry out experiments at different time points to evaluate the progression of electro-mechanical dysfunction in obesity and diabesity. Future experiments might include action potential experiments in individual myocytes from different regions of the heart, and at different ages, to assess the progress of obesity and diabesity on the electrophysiology of the heart. In addition, experiments might also include whole cell patch clamp experiments in ventricular myocytes to investigate other ionic currents, including Na^+^ and K^+^ currents, which are fundamental for the generation of action potentials. Imaging techniques including CT, MRI and echocardiography could be used to further understand the hemodynamic changes associated with obesity and diabesity. 

### 5.2. Clinical Impilcations

Our research demonstrates that ageing and prolonged diabesity lead to adverse clinical outcomes. It is important to implement strategies that will prevent disease progression, such as lifestyle changes—including exercise training and improved diet—and therapeutic interventions.

## Figures and Tables

**Table 1:** General characteristics of Zucker rats.

	ZL	ZF	ZDF
Body weight (g)	429.67 ± 9.43 (18) [11.61]	715.67 ± 13.81 (21) [44.52] *	601.86 ± 32.63 (21) [32.67] *
Heart weight (g)	1.7 ± 0.07 (18) [16.83]	2.2 ± 0.06 (21) [40.07] *	2.1 ± 0.08 (21) [32.64] *
Non-fasting blood Glucose (mg/dL)	118.47 ± 4.18 (17) [14.21]	136.67 ± 4.38 (21) [23.98]	345.10 ± 25.16 (21) [48.81] *#
Heart weight/body weight ratio (mg/g)	2.54 ± 0.09 (18) [17.56]	3.26 ± 0.11 (21) [43.67] *	2.84 ± 0.06 (21) [28.43] #
Plasma Insulin (µg/L)	2.09 ± 0.57 (10) [8.25]	12.70 ± 1.15 (9) [23.0] *	2.14 ± 0.29 (8) [11.06] #

Data are mean ± SEM, number of animals is in parenthesis and the mean rank is in brackets. One-way ANOVA followed by Bonferroni corrected *t* tests for multiple comparisons, * *p* < 0.05 compared to ZL, # *p* < 0.05 compared to ZF.

## Data Availability

The data presented in this study are available on request from the corresponding author.
